# Assessing the carbon footprint of egg production from white and brown laying hens under a no-culling male chick system: a Dutch case study

**DOI:** 10.1016/j.psj.2026.107290

**Published:** 2026-06-12

**Authors:** Seyyed Hassan Pishgar-Komleh, Pim Frederik Mostert

**Affiliations:** Wageningen Livestock Research, Wageningen University and Research, De Elst 1, Wageningen 6708 WD, the Netherlands

**Keywords:** Life cycle assessment, Carbon footprint, Egg, No-culling male chick, White/brown hen

## Abstract

Producing more ethical and animal-welfare friendly eggs is a public demand. This study was carried out to provide more insight into the environmental impacts of no-culling of male chicks (NCMC) in a laying hen (white and brown breeds) production system in the Netherlands. A life cycle assessment was conducted and three phases including i) the rearing and laying periods of parent stocks, ii) the hatchery stage, and iii) the rearing and laying periods of hens, were assessed. Based on the obtained results, the carbon footprint (CF) of market egg equipped with NCMC was 2.31 and 2.74 kg CO_2_eq/kg eggs for white and brown breeds, respectively . Feed production and land use change were the main contributors to the total greenhouse gas (GHG) emissions of parent stocks and hens phases. According to our findings, application of NCMC technique increased GHG emissions at hatchery stage by 98 and 56% for white and brown breeds. However, use of NCMC led to less than 1% higher CF of market eggs at the end of laying period of hens 1%. The higher GHG emissions were due to i) higher energy consumption, ii) higher egg losses, iii) higher errors in the sex determination process, and iv) lower hatchability. This modest rise in emissions showed that more animal friendly and ethical production can be achieved through the implementation of NCMC with minimal environmental trade-offs. Integrating such practices with improved feed techniques may offset the extra emissions while delivering higher sustainability outcome in overall. Comparison of different allocation methods demonstrated that the choice of allocation method influences the estimated effect of implementing the NCMC strategy on the CF of market eggs.

## Introduction

The global demand for animal protein is growing due to population growth. The poultry egg is an important source of protein globally to meet the growing demand of our ever-expanding world population. The global egg production is showing continued growth, reaching 91 million tons in 2023 (FAO, 2016), estimated to reach 95.6 million tons in 2030 (OECD, 2025). The egg production has the lowest carbon footprint **(CF)** per kg protein among various animal protein sources such as beef, lamb, veal, milk, cheese and pork highlighting its potential environmental advantages in the discussions of sustainable protein sources (Gaillac and Marbach, 2021; Poore and Nemecek, 2018). However, due to growing production, it is crucial to assess and reduce emissions from egg production to improve sustainability and align with the global climate goals.

Life Cycle Assessment **(LCA)** is a widely recognized method for evaluating the environmental impacts of food production systems throughout the entire life cycle. By performing an LCA for egg production, producers can make informed decisions, resulting in higher efficiency, lower resource use and a reduction in greenhouse gas **(GHG)** emissions. Furthermore, LCA promotes transparency within the egg production sector. Former LCA studies have shown that among different sources of GHG emissions in egg production systems, feed production is the largest contributor, followed by manure management and energy use (Abín et al., 2018; Dekker, et al., 2011; Ghasempour and Ahmadi, 2016; Guðjónsdóttir, et al., 2025; Kheiralipour, et al., 2024; Mason, 2023; Payandeh, et al., 2025; Pelaracci, et al., 2024; Pelletier, 2017; Taylor, et al., 2014; Xin, et al., 2011). Recent improvements in feed formulations, breeding, housing technologies, and manure management practices have helped the sector to reduce GHG emissions and improve the sustainability of egg production (Kheiralipour et al., 2024; Mollenhorst and de Haas, 2019). The transition to more sustainable production involves not only tackling environmental challenges but also several socio-economic issues. During the last decades, male chicks of laying hens were killed because males do not lay eggs and are not suitable to be reared for meat production. It is estimated that around 6-7 billion male chicks are killed annually worldwide (Krautwald-Junghanns, et al., 2018). Based on estimations, the number of male chicks killed in EU is around 230 million per year (EUROSTAT, 2022). It has raised strong ethical concerns. Producing more ethical and animal-welfare friendly eggs is a public demand and are increasing around the world (Van Asselt, et al., 2015). Efforts have been initiated within EU to reduce the number of chicks culled. There is no EU-wide ban on the killing of day-old male chicks, however, the European Commission **(EC)** is looking into the issue as part of a broader review of EU animal welfare legislation (EPRS, 2022).

A report by van Niekerk (2025) provides useful information on the latest state of affairs regarding the killing of day-old male chicks. Several EU countries i.e. Austria, Belgium, Germany, France, Netherlands, etc. set a number of regulatory and policies to restrict or phase out the killing of day-old male chicks (Coppola, et al., 2024; van Niekerk, 2025). Some of the main possible options to minimize the killing of male chicks are: i) development of dual-purpose breeds where females are raised as layers and males for meat production, ii) raising male chicks for meat production, iii) in-ovo sexing of embryos, iv) identification of mechanisms that can distort the sex ratio in favor of female chicks (Bruijnis, et al., 2015; Coppola et al., 2024; Gautron, et al., 2021; Krautwald-Junghanns et al., 2018). Among the mentioned options, i and ii increase the cost of production and with high possibility increase the GHG emissions, which makes them less attractive to producers. In-ovo sexing of embryos is an advanced technique to identify the sex of fertilized eggs before hatching. Recently, this technique has gained attention and some studies have focused on the technical issues and developments of this technique. This technique can affect parameters such as hatchability, accuracy, energy use, capacity, GHG emissions. Coppola et al. (2024) studied various in-ovo approaches and found in-ovo sex determination as the main potential alternative method to male chicks culling. To our knowledge, no study has evaluated the environmental impacts of applying the in-ovo sexing technique in egg production (Corion, et al., 2023; Jia, et al., 2023; Xie, et al., 2023).

To provide more insight into the environmental impacts of no-culling of male chicks (NCMC) on various egg production systems namely white and brown layer systems, an LCA was carried out. The goal of this study is to estimate whether NCMC, as a strategy to improve animal welfare aspect, results in higher GHG emissions of market eggs.

## Materials and methods

### Life cycle assessment – goal and scope

**Fig. 1:**
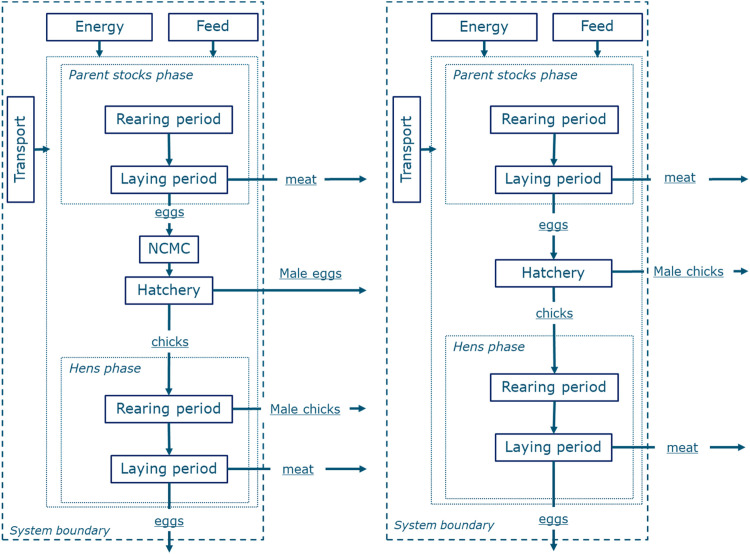
System boundaries and life cycle stages for egg production systems with (left) and without (right) No-Culling Male Chicks (NCMC).

In this study we applied an attributional LCA to calculate the impact of NCMC system on the total GHG emissions of produced eggs for two different breeds. H&N Super Nick and H&N Brown Nick were considered as white and brown breeds. A "cradle to farm gate" LCA was performed to calculate GHG emissions from the extraction and processing of raw materials to the production of market eggs at the farm gate. The system boundary for both white and brown laying hens and production systems with and without NCMC are shown in Fig. 1. The egg production system can be divided to breeding flocks, reproduction flocks (parent stocks), hatchery, rearing of laying hens, and egg production. However, in this study we considered three phases including parent stock (rearing and laying), hatchery and hens (rearing and laying) phases. In each growing phase, both rearing and laying periods were included in the system boundaries. At each production phase, GHG emissions were calculated for the production and transport of animal feed, production and consumption of energy resources, and on-farm emissions related to manure management. All emissions after the farm gate, such as transport of produced eggs to the market, consumption and waste management, were not included. The intermediate transport including the transport of feed ingredients and produced eggs (to NCMC unit and hatchery) and one day-old chicks to the laying hens production phase were included. The process flow of the production system with NCMC differs from the conventional system (Fig. 1). The fertilized eggs are first pre-incubated, followed by in-egg sexing before day 13 of incubation, after which only the eggs with female embryo are further incubated and hatched. The GHG emissions were calculated at different production phases, including i) parent stock (the rearing and laying periods), ii) the hatchery stage, and iii) hens (the rearing and laying periods) (Fig. 1). Therefore, different functional units **(FUs)** were applied to express GHG emissions at different production phases namely i) one hen for the rearing period of parent stocks and hens phases, ii) one delivered hatching egg for the laying period of parent stocks, iii) one day-old chick for the hatchery phase, and iii) kg egg for the laying period of hens.

Since both eggs and meat are produced at the end of laying period (both parent stocks and hens phases), the emissions must be allocated to the produced egg and meat. No emissions were allocated to animal manure. For this purpose, economic allocation (as the most common allocation method) was applied, whereby emissions are allocated to each product (eggs and meat) based on their economic value (more information on economic allocation can be found in the Supplementary File, Section 1). In addition to economic allocation, several allocation methods exist which can have high impact on the CF of the final product. To provide a comprehensive overview, in addition to economic allocation, we tested other options including no allocation, mass (real weight and protein-based), and biophysical allocation. By testing different allocation methods it is also possible to assess the impact of using different allocation methods on environmental impacts of NCMC. In a situation with no allocation, the emissions at each production phase are allocated to the main or primary product. In the mass allocation, the emissions at the end of a process are allocated based on the products real or protein-based mass. The protein content of 0.12 kg protein per kg egg and 0.19 kg protein per kg meat were used in the protein-based allocation method (Caffa, et al., 2025; MyFoodData, 2025). For the biophysical allocation, the allocation fraction for division of emissions to egg and live weight was calculated based on the energy requirements for growth and egg production which were assumed to be 21.5 kJ metabolized energy per g LW and 12.1 kJ metabolized energy per g egg (CVB, 2018).

The emissions of the three most important GHGs were included: carbon dioxide (CO_2_) (mainly fossil fuels and transport), methane (CH_4_) (mainly manure) and nitrous oxide (N_2_O) (manure storage and application for crop production). Based on their Global Warming Potential (100 years) (1 for CO_2_, 27 for CH_4_, 273 for N_2_O) the emissions were aggregated expressed in kg CO_2_eq (IPCC, 2021).

### Data collection and analysis

Primary data, including production parameters (e.g. male-female ratio, weight gain, mortality, feed intake), diet composition and transportation distances, were collected based on the representative commercial production conditions in the Netherlands. Primary data were provided by a large commercial poultry company operating within the Dutch egg production sector. Due to confidentiality agreements and data protection requirements, the identity of the company and specific farm-level information remain anonymous throughout the manuscript. Due to complexity and variability of commercial egg production systems, not all primary data could be collected from a statistically representative number of farms or production cycles. Therefore, a combination of primary industry data, expert knowledge, and representative commercial assumptions was used to develop the life cycle inventory. The objective of this study was not to compare individual farms or to establish statistically representative benchmarks for specific breeds or production systems, but rather to evaluate the environmental impacts of implementing NCMC within realistic commercial settings. Therefore, the modeled systems should be interpreted as representative case studies rather than exact representations of all Dutch egg production systems. Details of the production parameters and production periods used in environmental assessment can be found in the Supplementary File. The following sections present the collected and applied data and the calculation rules for GHG emissions.

#### Feed production

For the parent stock (rearing and laying), seventeen diets were composed and for the laying phase (rearing and laying), nine diets were composed. The composition of feed ingredients in the diets for parent stock and laying hens for two breeds were provided by a commercial anonymous producer. Details can be found in the Supplementary File (Table SF2). For each diet, GHG emissions of the feeds were calculated using Agri-footprint 6.3 (Blonk Consultants, 2022). Agri-footprint 6.3 takes into account GHG emissions associated with the production of different feed ingredients. The GHG emissions of all input processes (energy, production and use of fertilizers, transport, feed mill, additional processing and drying) involved in the production of one kilogram of feed, up to the poultry farm have been included. Some feed ingredients which were present in small amounts in the diet were not included in the database, so no CF value was available. In that case, comparable products were selected or assumptions were made. In addition to emissions related to the production and processing of feed ingredients, emissions from land use change **(LUC)** were also included. For the sake of transparency, accuracy and comparability in environmental assessments, LUC effects are reported separately from GHG emissions from feed production. Feed ingredients can originate from different countries. Since the exact origin of the feeds was unknown, the average values for the import of raw materials into the Netherlands were used, as presented in the Agri-footprint 6.3 database. The average CF of different feed diets are presented in the Supplementary File.

#### Energy on the farm

The energy consumption on the poultry farm (lighting, ventilation, heating, etc.) was based on Kwantitatieve Informatie voor de Nederlandse Veehouderij (KWIN) 2024-2025 (KWIN, 2024). No data was available on differences between parent stocks and laying hens or between white and brown hens. Details on the applied data can be found in the Supplementary File. The background data, including CF of materials and energy sources, were obtained from ecoinvent version 3.11 (ECOINVENT, 2023), where the emission values related to the Netherlands were selected.

#### Manure storage on the farm

The emissions of manure management were calculated as the sum of CH₄ emissions, direct N_2_O and indirect N_2_O emissions (NH₃ and NOₓ). IPCC (2019) and the Dutch National Inventory Report (RIVM, 2024) were followed. Where data was available, IPCC calculation method was adapted to the Dutch situation. Details of applied equations can be found in the Supplementary File (Section 2.4).

#### No-culling male chicks

At the hatchery phase, fertilized eggs hatched, and one day-old chicks are delivered to the rearing period of hens phase. The NCMC refers to a practice in the poultry sector where male chicks are not killed after hatching. The NCMC system uses alternative methods to avoid killing male chicks. This system is known by different names in different countries like *Ohne Küken Töten* in Germany and *Zonder Kuikendoden* in the Netherlands. To avoid killing male chicks, some techniques including in-ovo sexing and dual purpose breed can be applied. In-ovo technique employs different approaches or methods such as genetic, optical, biological, physicochemical, and biotechnological ones. In this study sex determination was carried out using i) hyperspectral imaging, and ii) artificial intelligent **(AI)** driven magnetic resonance imaging **(MRI)** methods. Comparison of techniques was beyond the aim of this study.

To assess the environmental impacts of NCMC, two scenarios namely hatchery without and with NCMC were studied. Data on i) losses (in percentage) during the sex determination process, ii) the decrease in hatchability (in percentage) and iii) the additional energy and transport costs in a system with NCMC was collected. These type of data were provided based on real situation (more information in the Supplementary File, Table SF1).

## Results

The following sections present the results of GHG analysis of the egg production chain.

### Parent stocks – rearing and laying period

The results of GHG analysis at the end of laying period of parent stocks phase for white and brown breeds are shown in Table 1. Feed production and LUC were the most important sources of GHG emissions. The next largest source of GHG emissions was emissions from chickens supplied from rearing period. The comparison of GHG emissions among breeds revealed a slight higher total GHG emissions for white (0.171 kg CO_2_eq/hatching egg) than brown (0.167 kg CO_2_eq/hatching egg). Due to low total economic value of produced meat compared with total economic value of eggs, small amount of total GHG emissions attributed to the meat.

**Table 1:** Greenhouse gas emissions per delivered hatching egg (kg CO_2_eq/hatching egg) at the end of laying period of the parent stock for white and brown breeds.

Item	Unit	White	Brown
Parent stock rearing	kg CO_2_eq/hatching egg	0.019	0.020
Manure management	kg CO_2_eq/hatching egg	0.007	0.007
Feed production	kg CO_2_eq/hatching egg	0.078	0.074
Land use change	kg CO_2_eq/hatching egg	0.064	0.062
Material and energy use	kg CO_2_eq/hatching egg	0.004	0.003
Total with allocation to egg	kg CO_2_eq/hatching egg	0.171	0.166
Total without allocation to egg	kg CO_2_eq/hatching egg	0.171	0.167

### Hatchery without and with no-culling male chicks

Table 2 shows the results of comparing GHG emissions of two scenarios namely without and with NCMC. During the hatchery phase, two main sources of GHG emissions are the material and energy use and the upstream emissions of parent stock laying (Table 2). GHG emissions associated with parent stocks laying were higher than GHG emissions from the material and energy use. The comparison of breeds showed the higher total emissions of white than brown.

**Table 2:** Greenhouse gas emissions per delivered day-old chick (kg CO_2_eq/day-old chick) at the end of hatching period of fertilized eggs for white and brown breeds with and without No-Culling Male Chicks.

Item	Unit	With No-Culling Male Chicks		Without No-Culling Male Chicks	
		White	Brown	White	Brown
Parent stock laying	kg CO_2_eq/day-old chick	0.457	0.432	0.413	0.392
Material and energy use	kg CO_2_eq/day-old chick	0.574	0.342	0.109	0.106
Total	kg CO_2_eq/day-old chick	1.031	0.775	0.521	0.498

As it is shown in Table 2, the comparison of two scenarios with and without NCMC showed that hatchery with NCMC increased the total GHG emissions by 98% and 56% for white and brown breeds compared with a hatchery without NCMC. This higher GHG emissions are because of i) a higher energy consumption, ii) a higher egg losses, iii) a higher errors in the sex determination process, and iv) a lower hatchability. Although NCMC results in higher GHGs, this process prevents culling of male chicks by extracting male eggs and producing few number of male chicks.

### Hens phase without and with no-culling male chicks

Table 3 shows CF of market eggs and the contribution of various emission sources to total GHG emissions. Similar to the parent stock phase, feed production and LUC (feed) were the main sources of GHG emissions. The upstream emissions which are the emissions associated with the production of reared chickens was the third largest source of GHG emissions. Manure management and material and energy use had the smallest impact. CF of produced market egg was 2.71 kg CO_2_eq/kg egg for brown and 2.29 kg CO_2_eq/kg egg for white for a production system without NCMC. Higher feed consumption per produced egg for brown (0.14 kg per egg) than the white breeds (0.12 kg per egg) was the main reason.

**Table 3:** Greenhouse gas emissions per kg delivered market egg (kg CO2eq/kg egg) at the end the laying period of hens for white and brown breeds with and without No-Culling Male Chicks.

Item	Unit	With No-Culling Male Chicks		Without No-Culling Male Chicks	
		White	Brown	White	Brown
Hatchery	kg CO2eq/kg egg	0.290	0.365	0.271	0.346
Manure management	kg CO2eq/kg egg	0.087	0.100	0.087	0.100
Feed production	kg CO2eq/kg egg	1.033	1.206	1.033	1.206
Land use change	kg CO2eq/kg egg	0.830	0.970	0.830	0.970
Material and energy use	kg CO2eq/kg egg	0.073	0.083	0.073	0.083
Total with allocation to egg	kg CO2eq/kg egg	2.313	2.724	2.294	2.705
Total without allocation to egg	kg CO2eq/kg egg	2.323	2.738	2.304	2.719

Brown breed produced heavier eggs than the white breed, but the number of eggs per hen was lower. This resulted in a lower total kilogram egg produced compared to the white breeds. For more detailed information, the feed conversion ratio, the number of eggs produced per hen, and the average egg weight were compared. The feed conversion ratio of white and brown was 33.0 kg feed/kg LW, and 29.2 kg feed/kg LW, respectively. Total egg production per hen was 446 eggs for white, and 392 eggs for brown. The average egg weight was 63.0 g/egg for white, and 63.3 g/egg for brown. To provide more information, the results of GHG emissions at the end of rearing phase of hens period can be found in the Supplementary File.

The results for CF of eggs produced in a system with NCMC showed the slightly higher (less than 1%) GHG emissions of rearing chickens compared to a system without NCMC. The CF of market egg for white and brown breeds equipped with NCMC was 2.31 and 2.72 kg CO_2_eq/kg egg, respectively. Although the environmental impacts of NCMC at the hatchery stage was high, the impacts declined at the end of laying period of hens due to greater impact of other sources of emissions.

### Application of different allocation methods

**Fig. 2:**
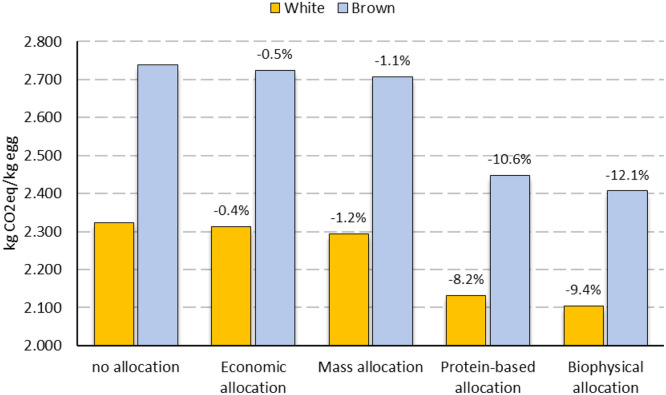
Carbon footprint (kg CO_2_eq/kg eggs) of market egg using different allocation method for production system with No-Culling Male Chick. Percentages on top of each column indicates the difference from no allocation.

**Fig. 3:**
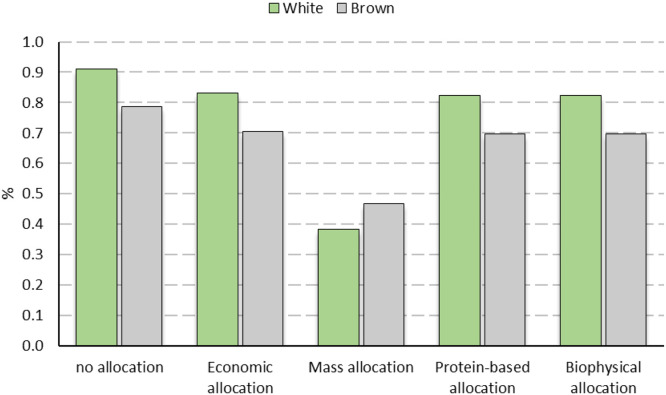
Impact of the No-Culling Male Chick on the carbon footprint of market eggs under different allocation methods. Results are presented as the percentage difference between systems with and without No-Culling Male Chick.

The results of assessing the impact of application of different allocation methods on CF of market egg under NCMC scenario for white and brown breeds are shown in Fig. 2, Fig. 3. The CFs for white and brown with no allocation were 2.32 and 2.74 kg CO_2_eq/kg eggs, respectively (Fig. 2). Compared to the no allocation situation, a reduction of 0.4%, 1.2%, 8.2% and 9.4% in CF of market egg of white was seen for economic, mass, protein-based and biophysical allocation, respectively. Application of economic, mass, protein-based and biophysical allocation resulted in the reduction of CF of market egg of brown breed by 0.5%, 1.1%, 10.6% and 12.1%, respectively. Obtained results illustrated that more than 99% of total GHG emissions are allocated to the market eggs by using economic and mass allocation. The protein-based allocation factor ranged from 89% to 92% across breeds and phases. The biophysical allocation factor varied between 88% and 91% across breeds and phase. To provide more detailed information, the results of allocation factor calculations are presented in the Supplementary File, Section 4.

The percentage difference in the CF of market eggs between systems with and without NCMC under different allocation methods for white and brown is shown in Fig. 3. The impact of NCMC varied considerably depending on the applied allocation method. For both breeds the largest differences were seen under no allocation method, followed by economic allocation, whereas mass allocation resulted in lower differences. The protein based and biophysical allocation methods produced similar differences. In general the percentage difference was higher for white breed than for the brown breed under all allocation methods except mas allocation, where the brown breed showed a greater difference.

## Discussion

Regardless of whether the NCMC was implemented, the CFs of market eggs ranged between 2.29 and 2.72 kg CO_2_eq/kg eggs across the evaluated scenario. These results were aligned with findings of Dekker et al. (2011) which showed a range of 2.23-2.75 for different egg production systems in the Netherlands. However, our finding was lower than CFs reported by Mollenhorst, et al. (2006) for various egg production systems in the Netherlands (3.9-4.6 kg CO_2_eq/kg eggs). Improvements on feed conversion rate is the reason for lower CFs in our study. Our findings on CF of market eggs was also similar to findings in the literature (ranged from 2.38 to 3.45 kg CO_2_eq/kg egg) for other countries (Abín et al., 2018; Guðjónsdóttir et al 2025; Guillaume, et al., 2022; Leinonen, et al., 2012; Pelletier, 2017; Turner, et al., 2022).

Feed production and LUC contributed most to the total GHG emissions of rearing and laying periods of parent stocks and hens phases, which was also found in other studies (Abín et al., 2018; Dekker et al., 2011; Ghasempour and Ahmadi, 2016). Maize, wheat and soybean were the main feed ingredients in our study and contributed to more than 70% of total mass of consumed feed. The origin of these feed ingredients has impact on their CF. Replacing the current feeds with the ones that require less transport, reduces the GHG emissions of diet. Moreover, some ingredients such as soybean can be replaced by other types of feed ingredient with lower CF such as legumes and beans (Baumgartner, et al., 2008). In a study conducted by Abín et al. (2018), soybean and palm oil were replaced with pea and cottonseed oil, resulted in reduction in CF of market eggs. Giannenas, et al. (2017) concluded that using corn gluten instead of soybean and adding protease could reduce environmental impacts of poultry feed without negative impact on animal performance.

Comparison of breeds showed a higher CF for brown eggs than the white breed due to higher feed consumption and upstream emissions. FCR was 2.2 kg feed per kg egg for brown while for white it was 1.9 kg feed per kg egg. Similar differences were reported by de Haas, et al. (2021). Feed conversion rate is a key parameter which affecting CF of market eggs (Dekker et al., 2011; Pelletier, et al., 2014) and that was also found in this study. Genetic selection of the laying hens, feed composition, and type of housing system are among the important items affecting feed conversion rate (Anderson, et al., 2013; de Haas et al., 2021). It was seen that brown breeds consumed more feed to gain more weight than the white breeds but produced less eggs which meant higher feed conversion (kg feed/kg egg) in total. One of items which has not been studied widely is the quality of feed ingredients. Over years, feed producers applied new techniques to produce crops with the higher quality (e.g. protein and fat content). This improvement is hidden in the feed conversion rate.

In the laying sector, millions of one day-old male chicks are culled because they can neither lay eggs nor produce meat comparable to broilers. One of the actions established by “Day-old male chicks” steering committee of the Netherlands government in 2023 was that no more day-old male chicks will be killed for market eggs for the Dutch and German markets in 2026 (Workamp, 2024). In-ovo sex determination, highlighted by (Coppola et al., 2024), is one the main potential alternative methods to male chicks culling. Moreover, it was the most preferred by consumers especially if performed during the first days of incubation (Coppola et al., 2024). The environmental impacts of in-ovo techniques which employed hyperspectral and combination of AI and MRI technology was assessed in our study and the obtained results showed less than 1% increase in the CF of markets egg due to application of NCMC. To the best of our knowledge no study focused on this assessing the impact of this system on GHG emissions of market eggs therefore it is not possible to compare our results with the findings of other research. The main reason of an increase in the GHG emissions of this system is greater energy consumption and greater losses in the sex determination process due to accuracy and effects on hatchability. Although the environmental impacts of NCMC was high at the hatchery stage, due to high contribution of other sources of emissions such as feed at the rearing and laying periods of hen pahse, the impact of NCMC is very low on the final CF of market eggs.

One of the aspects which has not been addressed in this study was the impact of NCMC on broader system or on the supply chain. In a system with culling, one day-old chicks are not entirely waste. Most of them are used as feed for zoo animals or processed into raw materials for pet food. Stopping male chick culling means other protein sources must replace them, such as meat by-products, insects, or specialized feed ingredients. Producing some of the alternative sources required resources and add to GHG emissions. Therefore, some of the burdens shifts elsewhere in the whole system. At the same time, male eggs produced in the no-culling system may enter the pet food market to replace the ingredients that used to come from other sources. It might replace crop protein source which results in reduction of GHG emissions. To sum it up, if the replacement proteins come from efficient and circular sources, a no-culling system can remain sustainable. If they come from new resource-intensive production, the environmental footprint may increase slightly, even if the ethical standard improves.

Besides the environmental impacts, economic aspect of this method is important. In a study conducted in the Netherlands in 2024, it was shown that hatcheries charge an additional €3.00 per reared pullet for in-ovo sexing in white-egg production. This is the higher purchase price for the laying hen farmer, who then spreads this cost over an average of 375 market eggs. Therefore, in-ovo sexing adds about €0.009 per market egg at farm level to the total cost of white eggs. At the packing stage the additional cost would be €0.011-0.0115 per market egg. For brown eggs, the increase is lower, about €0.008 per egg at the packing station. These values are averages and may vary with flock performance and packing-station efficiency (van Horne and Bondt, 2024).

On of the methodological challenges in the LCA of laying hen sector is application of allocation method. The choice between different allocation methods such as no-allocation, mass-based, economic-based, protein-based, biophysical-based, or system expansion allocation can significantly affect results, often leading to inconsistent or incomparable outcomes across studies. Our study showed that allocation method can have an impact on the impact of NCMC on the CF of market eggs.

As the limitation of this study and due to lack of data, some of inputs including disinfectant materials, bedding, medicines, machinery, buildings, and maintenance were excluded from GHG analysis, as their contribution is small. Packaging stage was out of the system boundary of this study.

## Conclusions

This study aimed to evaluate the impact of NCMC technique in a laying hen production system in the Netherlands. Therefore, two egg production system namely without and with NCMC were compared in terms of the total GHG emissions. Based on the obtained results, CF of market egg without using NCMC was 2.29 and 2.71 kg CO_2_eq/kg eggs for white and brown breeds. In the production system with NCMC the CF for white and breeds was 2.31 and 2.72 kg CO_2_eq/kg eggs, respectively. The main findings of this study was that NCMC increased the CF of market eggs by less than 1% in both white and brown breeds, even though GHG emissions at the hatchery stage increased substantially by 98% and 56%, respectively. This modest rise in emissions showed that more animal friendly and ethical production in laying hen sector can be achieved by minimal environmental trade-offs. As it was expected feed production and LUC were the main GHG contributors to the total GHG emissions of parent stocks and hens phases. Comparison of different allocation methods demonstrated that the choice of allocation method influences the estimated effect of implementing the NCMC strategy on the CF of market eggs. Overall, this research provides evidence that improving animal welfare through NCMC can be compatible with sustainability goals.

## Declaration of AI and AI-assisted technologies in the writing process

During the preparation of this work the author(s) used ChatGPT partially and only to improve readability and language of the paper. After using this tool/service, the author(s) reviewed and edited the content as needed and take(s) full responsibility for the content of the publication.

## CRediT authorship contribution statement

**Seyyed Hassan Pishgar-Komleh:** Writing – review & editing, Writing – original draft, Visualization, Validation, Software, Project administration, Methodology, Investigation, Formal analysis, Data curation, Conceptualization. **Pim Frederik Mostert:** Writing – review & editing, Methodology, Funding acquisition, Conceptualization.

## Disclosures

The authors declare that they have no known competing financial interests or personal relationships that could have appeared to influence the work reported in this paper.

## Appendix Supplementary materials

Image, application 1

## Footnotes

Supplementary material associated with this article can be found, in the online version, at doi:10.1016/j.psj.2026.107290.

## References

[bib0001] Abín R., Laca A., Laca A., Díaz M. (2018). Environmental assesment of intensive egg production: a Spanish case study. J. Clean. Prod..

[bib0002] Anderson K., Havenstein G., Jenkins P., Osborne J. (2013). Changes in commercial laying stock performance, 1958-2011: thirty-seven flocks of the North Carolina random sample and subsequent layerperformance and management tests. World's Poult. Sci. J..

[bib0003] Baumgartner, D., L. de Baan, and T. Nemecek. 2008. European grain legumes-environment-friendly animal feed? Life cycle assessement of pork, chicken meat, egg, and milk production. Final Report WP2 2.

[bib0004] Blonk Consultants (2022). https://www.agri-footprint.com.

[bib0005] Bruijnis M., Blok V., Stassen E., Gremmen H. (2015). Moral “Lock-In” in responsible innovation: the ethical and social aspects of killing day-old chicks and its alternatives. J. Agric. Environ. Ethics.

[bib0006] Caffa I., Proietti E., Turrini F., Borgarelli C., Ferrando M.R., Formisano E., de Cassya Lopes Neri L., Martini D., Angelino D., Tagliabue A. (2025). Nutritional aspects of eggs for a healthy and sustainable consumption: a narrative review. Food Sci. Nutr..

[bib0007] Coppola F., Paci G., Profeti M., Mancini S. (2024). Stop culling male layer-type chick: an overview of the alternatives and public perspective. Worlds Poult. Sci. J..

[bib0008] Corion, M., S. Santos, B. De Ketelaere, D. Spasic, M. Hertog, and J. Lammertyn. 2023. Insights and interpretation of the trends for in ovo sexing technologies in papers and patents. 10.1186/s40104-023-00898-1 PMC10347793 37452378

[bib0009] CVB. 2018. Tabellenboek Veevoeding 2018. Voedernormen Pluimvee en voederwaarden voedermiddelen voor Pluimvee. CVB-reeks nr. 60.

[bib0010] de Haas Y., Bink M.C., Borg R., Koenen E.P., Verschuren L.M., Mollenhorst H., Baines R. (2021). The Contribution of Animal Breeding to Reducing the Environmental Impact of Livestock Production. Pages 57-80 in Reducing Greenhouse Gas Emissions from Livestock Production.

[bib0011] Dekker S., De Boer I.J., Vermeij I., Aarnink A.J., Koerkamp P.G. (2011). Ecological and economic evaluation of Dutch egg production systems. Livest. Sci..

[bib0012] ECOINVENT (2023). https://www.ecoinvent.org.

[bib0013] EPRS (2022).

[bib0014] EUROSTAT (2022). Poultry statistics - statistics explained. https://ec.europa.eu/eurostat/statistics-explained/index.php?title=Poultry_statistics#Poultry.

[bib0015] FAO (2016). FAOSTAT database collections. http://faostat.fao.org.

[bib0016] Gaillac R., Marbach S. (2021). The carbon footprint of meat and dairy proteins: a practical perspective to guide low carbon footprint dietary choices. J. Clean. Prod..

[bib0017] Gautron J., Réhault-Godbert S., Van de Braak T., Dunn I. (2021). What are the challenges facing the table egg industry in the next decades and what can be done to address them?. Animal.

[bib0018] Ghasempour A., Ahmadi E. (2016). Assessment of environment impacts of egg production chain using life cycle assessment. J. Environ. Manag..

[bib0019] Giannenas I., Bonos E., Anestis V., Filioussis G., Papanastasiou D.K., Bartzanas T., Papaioannou N., Tzora A., Skoufos I. (2017). Effects of protease addition and replacement of soybean meal by corn gluten meal on the growth of broilers and on the environmental performances of a broiler production system in Greece. PLoS One.

[bib0020] Guðjónsdóttir S.B., Vásquez-Mejía C..M., Shrivastava S., Ögmundarson Ó. (2025). A life cycle assessment of broiler chicken meat and egg production in Iceland. Poult. Sci..

[bib0021] Guillaume A., Hubatová-Vacková A., Kočí V. (2022). Environmental impacts of egg production from a life cycle perspective. Agriculture.

[bib0022] Calvo Buendia E., Tanabe K., Kranjc A., Baasansuren J., Fukuda M., Ngarize S., Osako A., Pyrozhenko Y., Shermanau P., Federici S., IPCC (2019). 2019 Refinement to the 2006 IPCC Guidelines for National Greenhouse Gas Inventories.

[bib0023] IPCC (2021). https://www.ipcc.ch/report/ar6/wg1/.

[bib0024] Jia N., Li B., Zhu J., Wang H., Zhao Y., Zhao W. (2023). A review of key techniques for in ovo sexing of chicken eggs. Agriculture.

[bib0025] Kheiralipour K., Rafiee S., Karimi M., Nadimi M., Paliwal J. (2024). The environmental impacts of commercial poultry production systems using life cycle assessment: a review. Worlds Poult. Sci. J..

[bib0026] Krautwald-Junghanns M.-E., Cramer K., Fischer B., Förster A., Galli R., Kremer F., Mapesa E., Meissner S., Preisinger R., Preusse G. (2018). Current approaches to avoid the culling of day-old male chicks in the layer industry, with special reference to spectroscopic methods. Poult. Sci..

[bib0027] KWIN (2024). https://shop.wur.nl/kwin/kwin-veehouderij-2024-25.html.

[bib0028] Leinonen I., Williams A., Wiseman J., Guy J., Kyriazakis I. (2012). Predicting the environmental impacts of chicken systems in the United Kingdom through a life cycle assessment: egg production systems. Poult. Sci..

[bib0029] Mason P. (2023). The importance of eggs in an environmentally sustainable diet. Nutr. Bull..

[bib0030] Mollenhorst H., Berentsen P., De Boer I. (2006). On-farm quantification of sustainability indicators: an application to egg production systems. Br. Poult. Sci..

[bib0031] Mollenhorst H., de Haas Y. (2019).

[bib0032] MyFoodData. 2025. MyFoodData.Com. Last access: <20.10.2025>.

[bib0033] OECD (2025). OECD-FAO agricultural Outlook 2021-2030. https://stats.oecd.org.

[bib0034] Payandeh Z., Mesri-Gundoshmian T., Jahanbakhshi A., Shahgholi G.H., Rossi L., Bacenetti J. (2025). Optimization of environmental and energy performance of egg production using data envelopment analysis (DEA) and life cycle assessment (LCA). Sci. Total Environ..

[bib0035] Pelaracci S., Paolotti L., Rocchi L., Boggia A., Castellini C. (2024). Life cycle assessment of organic and conventional egg production: a case study in northern Italy. Clean. Environ. Syst..

[bib0036] Pelletier N. (2017). Life cycle assessment of Canadian egg products, with differentiation by hen housing system type. J. Clean. Prod..

[bib0037] Pelletier N., Ibarburu M., Xin H. (2014). Comparison of the environmental footprint of the egg industry in the United States in 1960 and 2010. Poult. Sci..

[bib0038] Poore J., Nemecek T. (2018). Reducing food’s environmental impacts through producers and consumers. Science (1979).

[bib0039] RIVM (2024).

[bib0040] Taylor R., Omed H., Edwards-Jones G. (2014). The greenhouse emissions footprint of free-range eggs. Poult. Sci..

[bib0041] Turner I., Heidari D., Pelletier N. (2022). Life cycle assessment of contemporary Canadian egg production systems during the transition from conventional cage to alternative housing systems: update and analysis of trends and conditions. Resour. Conserv. Recycl..

[bib0042] Van Asselt E., Van Bussel L., Van Horne P., Van der Voet H., Van der Heijden G., Van der Fels-Klerx H. (2015). Assessing the sustainability of egg production systems in The Netherlands. Poult. Sci..

[bib0043] van Horne, P., and N. Bondt. 2024. Meerkosten uitfaseren doden eendagshaantjes.

[bib0044] van Niekerk T. (2025).

[bib0045] Workamp J. (2024). Roadmap Uitfaseren van het doden van eendagshaantjes van legrassen. “day-old male chicks” steering committee of the Netehrland government. https://open.overheid.nl/documenten/b8fdcd79-ee34-4921-a7a6-bc488d74d0dd/file.

[bib0046] Xie C., Tang W., Yang C. (2023). A review of the recent advances for the in-ovo sexing of chicken embryos using optical sensing techniques. Poult. Sci..

[bib0047] Xin H., Gates R.S., Green A.R., Mitloehner F.M., Moore P.A., Wathes C. (2011). Environmental impacts and sustainability of egg production systems. Poult. Sci..

