# Supplementary material for: Assessing the carbon footprint of egg production from white and brown laying hens under a no-culling male chick system: a Dutch case study
Source: Poult Sci. 2026 Jun 12;105(10):107290. doi: 10.1016/j.psj.2026.107290 (PMC13316583; doi:10.1016/j.psj.2026.107290)
Supplement: Supplementary file 1 [file mmc1.docx]

*Supplementary File*

**Assessing the carbon footprint of egg production from white and brown laying hens under a no-culling male chick system: a Dutch case study**

Seyyed Hassan Pishgar-Komleh^1*^, Pim Frederik Mostert^*^

* Wageningen Livestock Research, Wageningen University and Research

^1^ Corresponding author: Seyyed Hassan Pishgar-Komleh, De Elst 1, 6708 WD Wageningen

Email: [hassan.pishgarkomleh@wur.nl](mailto:hassan.pishgarkomleh@wur.nl)

Phone: +3131 7481 608

This document provides supporting information to the original manuscript. The *Supplementary* *File* includes details on economic allocation, primary data, greenhouse gas (GHG) calculation method, carbon footprint of diets, and the results on GHG analysis and application of different allocation methods.

# ECONOMIC ALLOCATION

As it has been stated in the manuscript, economic allocation was the primary allocation method. All price data used in this study are representative of the Netherlands. In the laying period of parent stocks phase, the price of a fertilized egg and an old chicken (for meat purposes) was assumed to be €0.19 per egg and €0.15 per kg live weight, respectively, based on the real market prices in 2025. The same prices were used for all studied breeds.

In the hatchery phase, the prices of day-old chick was €0.80 and €0.76 per chick for white and brown breeds (KWIN, 2024). Prices of chicks were significantly higher after application of the sex determination method (no-culling male chicks), namely €3.60 and €2.36 per female chick for white and brown breeds, respectively (KWIN, 2024). Based on the information from egg producers, no price was given to the day-old male chicks and eggs sent to the zoo and pet food industry. In the hens phase, values ​​of €0.13 and €0.14 per egg were used for white and brown breeds, respectively in the basis of the real market data. The price of an old hen at the end of the laying period (for meat purposes) was €0.15 per kg live weight for all breeds.

# DATA COLLECTION

## Production related parameters

Primary data, including production parameters (e.g. male-female ratio), animal performance (weight gain, mortality, feed intake), feed consumption and transportation distances, were collected based on real farm data. Table SF1 shows the details of the production parameters for different production phases and breeds, used in GHG calculations.

Table SF1 Primary data, including production parameters, animal performance, feed consumption and transportation distances of different production phases for white and brown laying hen breeds.

| Production phase | Item | Unit | White | Brown |
| --- | --- | --- | --- | --- |
| Rearing period – parent stock | Number of hens per rooster | hens/rooster | 14.3 | 12.5 |
|  | Number of hens at start | hens/m² | 11 | 11 |
|  | Flock size | bird/farm | 44,000 | 44,000 |
|  | Housing system | - | Litter floor | Litter floor |
|  | Mortality rate hens | % | 4.1 | 3.5 |
|  | Mortality rate roosters | % | 6.4 | 4.5 |
|  | Weight of hen at start (day 1) | kg/pullet | 0.04 | 0.04 |
|  | Weight of rooster at start (day 1) | kg/cockerel | 0.04 | 0.04 |
|  | Weight of hen at end | kg/hen | 1.25 | 1.45 |
|  | Weight of rooster at end | kg/rooster | 1.63 | 2.05 |
|  | Length of rearing period | days | 119 | 119 |
|  | Empty period | days | 21 | 21 |
|  | Feed intake | kg/delivered animals | 5.8 | 6.1 |
|  | Feed conversion | kg feed/kg body weight | 4.55 | 4.08 |
|  | Electricity consumption | kWh/delivered animals | 0.97 | 0.96 |
|  | Water consumption | m³/delivered animals | 0.02 | 0.02 |
|  | Heating consumption | m³/delivered animals | 0.13 | 0.13 |
| Laying period – parent stock | Number of hens per rooster | hens/rooster | 15 | 13 |
|  | Number of hens at start | hens/m² | 9 | 9 |
|  | Flock size | bird/farm | 40,000 | 40,000 |
|  | Housing system | - | Litter floor | Litter floor |
|  | Mortality rate hens | % | 5.0 | 6.8 |
|  | Mortality rate roosters | % | 13.5 | 12.6 |
|  | Weight of hen at end | kg/hen | 1.7 | 2.0 |
|  | Weight of rooster at end | kg/rooster | 2.3 | 2.9 |
|  | Length of laying period | days | 441 | 476 |
|  | Empty period | days | 21 | 21 |
|  | Feed intake | kg/delivered animals | 48.5 | 50.5 |
|  | Feed conversion | kg feed/kg Live weight | 30.16 | 26.68 |
|  | Eggs consumed per hen | egg/hen | 0 | 0 |
|  | Total egg production for hatchery | egg/hen | 362 | 383 |
|  | Average egg weight | g/egg | 60.5 | 58.5 |
|  | Egg loss | % | 1 | 1 |
|  | Electricity consumption | kWh/delivered animals | 1.89 | 1.93 |
|  | Water consumption | m³/delivered animals | 0.09 | 0.10 |
|  | Heating consumption | m³/delivered animals | 0.00 | 0.00 |
| Hatchery without no-culling male chicks | Average hatchability (female chicks) | % | 41.4 | 42.5 |
|  | Transport distance (farm to hatchery) | km | 200 | 200 |
|  | Electricity consumption | kWh/egg | 0.00 | 0.00 |
|  | Heating consumption | m³/egg | 0.02 | 0.02 |
| Hatchery with no-culling male chicks | Average hatchability (female chicks) | % | 37.4 | 38.5 |
|  | Transport distance (farm to hatchery) | km | 329 | 200 |
|  | Electricity consumption | kWh/egg | 0.11 | 0.00 |
|  | Heating consumption | m³/egg | 0.02 | 0.02 |
| Rearing period – laying hens | Number of hens at start | hens/m² | 11 | 11 |
|  | Flock size | bird/farm | 82,000 | 82,000 |
|  | Housing system | - | Litter floor | Litter floor |
|  | Mortality rate hens | % | 3.45 | 2.27 |
|  | Weight of hen at start | kg/animal | 0.04 | 0.04 |
|  | Weight of hen at end | kg/animal | 1.23 | 1.42 |
|  | Length of rearing period | days | 119 | 119 |
|  | Empty period | days | 21 | 21 |
|  | Sexing error (with NCMC^1^) | % | 0.4 | 0 |
|  | Sexing error (without NCMC^1^) | % | 1.0 | 3.5 |
|  | Feed intake without NCMC^1^ | kg/delivered animals | 6.80 | 7.80 |
|  | Feed intake with NCMC^1^ | kg/delivered animals | 6.81 | 7.99 |
|  | Feed conversion without NCMC^1^ | kg feed/kg body weight | 5.51 | 5.51 |
|  | Feed conversion with NCMC^1^ | kg feed/kg body weight | 5.52 | 5.65 |
|  | Electricity consumption without NCMC^1^ | kWh/delivered animals | 0.96 | 0.95 |
|  | Water consumption without NCMC^1^ | m³/delivered animals | 0.02 | 0.02 |
|  | Heating consumption without NCMC^1^ | m³/delivered animals | 0.13 | 0.13 |
|  | Electricity consumption with NCMC^1^ | kWh/delivered animals | 0.96 | 0.97 |
|  | Water consumption with NCMC^1^ | m³/delivered animals | 0.02 | 0.02 |
|  | Heating consumption with NCMC^1^ | m³/delivered animals | 0.13 | 0.14 |
| Laying period – laying hens | Number of hens at start | hens/m² | 9 | 9 |
|  | Flock size | bird/farm | 80,000 | 80,000 |
|  | Housing system | - | Litter floor | Litter floor |
|  | Mortality rate hens | % | 8.8 | 7.6 |
|  | Weight of hen at end | kg/hen | 1.79 | 2.06 |
|  | Length of laying period | days | 476 | 476 |
|  | Empty period | days | 21 | 21 |
|  | Feed intake | kg/starting animals (rooster + hen) | 53.8 | 55.5 |
|  | Feed conversion | kg feed/kg body weight | 32.95 | 29.15 |
|  | Eggs consumed per hen | egg/hen | 0 | 0 |
|  | Total egg production | egg/hen | 446 | 392 |
|  | Average egg weight | g/egg | 63.0 | 63.3 |
|  | Egg loss | % | 0 | 0 |
|  | Electricity consumption | kWh/delivered animals | 3.05 | 3.01 |
|  | Water consumption | m³/delivered animals | 0.10 | 0.10 |
|  | Heating consumption | m³/delivered animals | 0.00 | 0.00 |

^1 no-culling male chicks (NCMC)^

## Feed production

Diet composition for parent stock and laying hens for the studied breeds were provided by an anonymous producer. Details can be found in Table SF2. For each diet, the GHG emissions associated with the production of feeds were calculated using Agri-footprint 6.3 (Blonk Consultants, 2022). The GHG emissions of all input processes including energy, production and use of fertilizers, transport, feed mill, additional processing and drying were involved in the production of one kilogram of feed up to the laying hen farm. Due to the unavailability of specific carbon footprint (CF) values for certain feed ingredients, proxy values from compositionally or functionally similar feedstuffs were used. These substitutions were made for ingredients whose contribution to the total diet was minimal. This approach aligns with common practice in life cycle assessment (LCA) when primary data are lacking, and is not expected to significantly affect the robustness of the results. As it has been mentioned in the manuscript, feed ingredients can originate from different countries. Since the exact origin of the feeds was unknown, the average values ​​for the import of raw materials in the Netherlands were used. The average CF of different feed diets is shown in Table SF3.

Table SF2 Diet composition per production phase and period for different breeds.

| Ingredient | Parents stock | | | | |  | | | |  | | | |  | | | | Hens | | | |  | | |  |  |
| --- | --- | --- | --- | --- | --- | --- | --- | --- | --- | --- | --- | --- | --- | --- | --- | --- | --- | --- | --- | --- | --- | --- | --- | --- | --- | --- |
|  | Rearing | | | | |  | | | | Laying | | | |  | | | | Rearing | | | |  | | | Laying |  |
|  | White | | | | | Brown | | | | White | | | | Brown | | | | White | | | | Brown | | | White | Brown |
|  | Share in diet (%) | | | | |  | | | |  | | | |  | | | |  | | | |  | | |  |  |
| Diet code | 151914 | 151915 | 151910 | 151911 | 151912 | 151914 | 151915 | 151916 | 151917 | 163201 | 163221 | 163231 | 163241 | 163202 | 163222 | 163232 | 163242 | 151030 | 151050 | 151100 | 151200 | 151010 | 151100 | 151200 | 170000 | 170000 |
| Maize | 32.9 | 32.5 | 32.5 | 32.5 | 31.9 | 32.9 | 32.5 | 30.4 | 31.2 | 32.5 | 32.5 | 32.5 | 30.8 | 34.3 | 32.5 | 32.5 | 30.9 | 32.5 | 32.5 | 31.5 | 31.9 | 32.5 | 31.5 | 31.9 | 22.3 | 22.3 |
| Barley | 3.0 | 3.0 | 3.0 | 3.0 | 3.0 | 3.0 | 3.0 | 3.0 | 4.0 | 3.0 | 3.0 | 3.0 | 3.0 | 0.0 | 0.0 | 0.0 | 0.0 | 3.0 | 3.0 | 3.0 | 3.0 | 3.0 | 3.0 | 3.0 | 2.4 | 2.4 |
| Peas | 0.0 | 0.0 | 0.0 | 0.0 | 0.0 | 0.0 | 0.0 | 0.0 | 0.0 | 3.5 | 3.5 | 3.5 | 2.5 | 2.5 | 3.5 | 3.5 | 2.5 | 0.0 | 0.0 | 0.0 | 0.0 | 0.0 | 0.0 | 0.0 | 2.0 | 2.0 |
| Wheat | 18.6 | 22.6 | 23.1 | 25.3 | 31.6 | 18.6 | 22.6 | 26.5 | 30.9 | 24.5 | 22.4 | 22.1 | 24.8 | 26.0 | 23.2 | 23.4 | 27.5 | 21.1 | 23.3 | 25.0 | 30.6 | 21.1 | 25.0 | 30.6 | 30.0 | 30.0 |
| Wheat Gluten Feed | 7.5 | 4.0 | 4.0 | 4.0 | 2.4 | 7.5 | 4.0 | 0.0 | 0.0 | 4.0 | 1.5 | 3.3 | 2.5 | 0.0 | 0.0 | 0.0 | 0.0 | 0.0 | 4.0 | 4.0 | 4.0 | 0.0 | 4.0 | 4.0 | 0.0 | 0.0 |
| Wheat Grits | 0.0 | 0.0 | 0.0 | 0.0 | 1.6 | 0.0 | 0.0 | 4.0 | 4.0 | 0.0 | 0.0 | 0.0 | 0.0 | 3.5 | 2.0 | 0.5 | 1.5 | 7.4 | 0.0 | 0.0 | 0.0 | 7.4 | 0.0 | 0.0 | 0.0 | 0.0 |
| Lecithin/Fatty Acids | 0.0 | 0.0 | 0.0 | 0.0 | 0.0 | 0.0 | 0.0 | 0.0 | 0.0 | 0.0 | 0.0 | 0.0 | 0.0 | 0.0 | 0.0 | 0.0 | 0.0 | 0.0 | 0.0 | 0.3 | 0.3 | 0.0 | 0.3 | 0.3 | 0.0 | 0.0 |
| Soybean Oil | 1.5 | 0.8 | 1.0 | 0.8 | 0.8 | 1.5 | 0.8 | 0.8 | 0.8 | 1.3 | 2.2 | 1.9 | 2.5 | 0.0 | 1.6 | 1.6 | 1.5 | 1.5 | 0.8 | 0.5 | 0.5 | 1.5 | 0.5 | 0.5 | 1.0 | 1.0 |
| Fish Oil High in Omega-3 | 0.0 | 0.0 | 0.0 | 0.0 | 0.0 | 0.0 | 0.0 | 0.0 | 0.0 | 0.0 | 0.0 | 0.5 | 0.0 | 0.0 | 0.0 | 0.0 | 0.0 | 0.0 | 0.0 | 0.0 | 0.0 | 0.0 | 0.0 | 0.0 | 0.0 | 0.0 |
| Poultry Fat | 0.0 | 0.0 | 0.0 | 0.0 | 0.0 | 0.0 | 0.0 | 0.0 | 0.0 | 0.0 | 0.0 | 0.0 | 0.0 | 0.7 | 0.5 | 0.6 | 0.5 | 0.0 | 0.0 | 0.0 | 0.0 | 0.0 | 0.0 | 0.0 | 2.1 | 2.1 |
| Functional Fiber | 1.5 | 1.0 | 1.0 | 1.0 | 1.0 | 1.5 | 1.0 | 1.0 | 1.0 | 1.5 | 1.5 | 1.0 | 0.5 | 1.5 | 1.5 | 1.5 | 1.0 | 1.5 | 1.0 | 1.5 | 1.5 | 1.5 | 1.5 | 1.5 | 0.0 | 0.0 |
| Soy Hulls | 0.0 | 0.0 | 0.0 | 0.0 | 0.0 | 0.0 | 0.0 | 0.0 | 0.0 | 0.0 | 0.0 | 0.0 | 0.0 | 0.0 | 0.0 | 0.0 | 0.0 | 0.0 | 0.5 | 0.8 | 0.0 | 0.0 | 0.8 | 0.0 | 0.0 | 0.0 |
| High-Protein Soybean Meal | 23.5 | 20.0 | 17.8 | 13.2 | 3.0 | 23.5 | 20.0 | 15.6 | 5.9 | 10.2 | 13.0 | 12.3 | 12.7 | 6.5 | 13.5 | 12.7 | 12.6 | 22.0 | 19.9 | 13.5 | 4.2 | 22.0 | 13.5 | 4.2 | 11.8 | 11.8 |
| Rapeseed | 1.8 | 2.8 | 3.7 | 4.9 | 9.2 | 1.8 | 2.8 | 2.9 | 6.8 | 0.0 | 0.0 | 0.0 | 0.0 | 5.0 | 1.0 | 1.0 | 1.0 | 2.5 | 3.2 | 6.5 | 8.0 | 2.5 | 6.5 | 8.0 | 6.1 | 6.1 |
| Sunflower Seed Meal | 5.0 | 9.5 | 10.0 | 11.5 | 12.5 | 5.0 | 9.5 | 12.0 | 12.5 | 13.2 | 11.0 | 10.0 | 10.0 | 13.5 | 11.0 | 12.3 | 9.8 | 4.0 | 8.5 | 10.0 | 13.5 | 4.0 | 10.0 | 13.5 | 5.3 | 5.3 |
| Potato Protein | 0.5 | 0.0 | 0.0 | 0.0 | 0.0 | 0.5 | 0.0 | 0.0 | 0.0 | 0.0 | 0.0 | 0.0 | 0.0 | 0.0 | 0.0 | 0.0 | 0.0 | 0.5 | 0.0 | 0.0 | 0.0 | 0.5 | 0.0 | 0.0 | 0.0 | 0.0 |
| Monocalcium Phosphate | 0.0 | 0.0 | 0.0 | 0.0 | 0.0 | 0.0 | 0.0 | 0.0 | 0.0 | 0.0 | 0.0 | 0.0 | 0.0 | 0.0 | 0.0 | 0.0 | 0.0 | 1.0 | 0.0 | 0.0 | 0.0 | 1.0 | 0.0 | 0.0 | 0.5 | 0.5 |
| Dicalcium Phosphate | 1.2 | 1.0 | 1.0 | 1.0 | 0.7 | 1.2 | 1.0 | 1.0 | 0.6 | 0.4 | 0.6 | 0.5 | 0.3 | 0.4 | 0.6 | 0.5 | 0.4 | 0.0 | 1.0 | 1.0 | 0.6 | 0.0 | 1.0 | 0.6 | 0.0 | 0.0 |
| Coarse Limestone | 0.0 | 0.0 | 0.0 | 0.0 | 0.0 | 0.0 | 0.0 | 0.0 | 0.0 | 4.3 | 7.0 | 7.9 | 8.8 | 4.2 | 7.2 | 8.2 | 9.2 | 0.0 | 0.0 | 0.0 | 0.0 | 0.0 | 0.0 | 0.0 | 7.7 | 7.7 |
| Chalk | 1.2 | 1.1 | 0.8 | 0.9 | 0.7 | 1.2 | 1.1 | 0.9 | 0.8 | 0.0 | 0.0 | 0.0 | 0.0 | 0.0 | 0.0 | 0.0 | 0.0 | 1.4 | 1.1 | 0.9 | 0.7 | 1.4 | 0.9 | 0.7 | 2.0 | 2.0 |
| CleanActive 500 (likely a commercial additive) | 0.0 | 0.0 | 0.0 | 0.0 | 0.0 | 0.0 | 0.0 | 0.0 | 0.0 | 0.1 | 0.1 | 0.0 | 0.0 | 0.1 | 0.1 | 1.2 | 1.1 | 0.0 | 0.0 | 0.0 | 0.0 | 0.0 | 0.0 | 0.0 | 0.0 | 0.0 |
| Vitamins/Minerals premix | 1.6 | 1.4 | 1.7 | 1.5 | 1.3 | 1.6 | 1.4 | 1.6 | 1.2 | 1.1 | 1.3 | 1.2 | 1.2 | 1.3 | 1.3 | 0.1 | 0.1 | 1.6 | 1.3 | 1.5 | 1.3 | 1.6 | 1.5 | 1.3 | 0.0 | 0.0 |
| Canthaxanthin 0.5% premix | 0.0 | 0.0 | 0.0 | 0.0 | 0.0 | 0.0 | 0.0 | 0.0 | 0.0 | 0.1 | 0.1 | 0.1 | 0.1 | 0.1 | 0.1 | 0.4 | 0.4 | 0.0 | 0.0 | 0.0 | 0.0 | 0.0 | 0.0 | 0.0 | 0.0 | 0.0 |
| Oregano | 0.1 | 0.0 | 0.0 | 0.0 | 0.0 | 0.1 | 0.0 | 0.0 | 0.0 | 0.0 | 0.0 | 0.0 | 0.0 | 0.0 | 0.0 | 0.0 | 0.0 | 0.0 | 0.0 | 0.0 | 0.0 | 0.0 | 0.0 | 0.0 | 0.0 | 0.0 |
| Salmonella Killer (antimicrobial additive) | 0.2 | 0.4 | 0.4 | 0.4 | 0.4 | 0.2 | 0.4 | 0.4 | 0.4 | 0.4 | 0.4 | 0.4 | 0.4 | 0.4 | 0.4 | 0.0 | 0.0 | 0.0 | 0.0 | 0.0 | 0.0 | 0.0 | 0.0 | 0.0 | 0.0 | 0.0 |
| Biscuit Meal, Crude Fat<120 g/kg | 0.0 | 0.0 | 0.0 | 0.0 | 0.0 | 0.0 | 0.0 | 0.0 | 0.0 | 0.0 | 0.0 | 0.0 | 0.0 | 0.0 | 0.0 | 0.0 | 0.0 | 0.0 | 0.0 | 0.0 | 0.0 | 0.0 | 0.0 | 0.0 | 1.0 | 1.0 |
| Bread Meal | 0.0 | 0.0 | 0.0 | 0.0 | 0.0 | 0.0 | 0.0 | 0.0 | 0.0 | 0.0 | 0.0 | 0.0 | 0.0 | 0.0 | 0.0 | 0.0 | 0.0 | 0.0 | 0.0 | 0.0 | 0.0 | 0.0 | 0.0 | 0.0 | 1.0 | 1.0 |
| DL-Methionine | 0.0 | 0.0 | 0.0 | 0.0 | 0.0 | 0.0 | 0.0 | 0.0 | 0.0 | 0.0 | 0.0 | 0.0 | 0.0 | 0.0 | 0.0 | 0.0 | 0.0 | 0.0 | 0.0 | 0.0 | 0.0 | 0.0 | 0.0 | 0.0 | 0.1 | 0.1 |
| Phytase | 0.0 | 0.0 | 0.0 | 0.0 | 0.0 | 0.0 | 0.0 | 0.0 | 0.0 | 0.0 | 0.0 | 0.0 | 0.0 | 0.0 | 0.0 | 0.0 | 0.0 | 0.0 | 0.0 | 0.0 | 0.0 | 0.0 | 0.0 | 0.0 | 0.3 | 0.3 |
| Oats | 0.0 | 0.0 | 0.0 | 0.0 | 0.0 | 0.0 | 0.0 | 0.0 | 0.0 | 0.0 | 0.0 | 0.0 | 0.0 | 0.0 | 0.0 | 0.0 | 0.0 | 0.0 | 0.0 | 0.0 | 0.0 | 0.0 | 0.0 | 0.0 | 2.0 | 2.0 |
| Mervit Rearing 2849 (likely a commercial premix) | 0.0 | 0.0 | 0.0 | 0.0 | 0.0 | 0.0 | 0.0 | 0.0 | 0.0 | 0.0 | 0.0 | 0.0 | 0.0 | 0.0 | 0.0 | 0.0 | 0.0 | 0.0 | 0.0 | 0.0 | 0.0 | 0.0 | 0.0 | 0.0 | 0.5 | 0.5 |
| Sodium Bicarbonate | 0.0 | 0.0 | 0.0 | 0.0 | 0.0 | 0.0 | 0.0 | 0.0 | 0.0 | 0.0 | 0.0 | 0.0 | 0.0 | 0.0 | 0.0 | 0.0 | 0.0 | 0.0 | 0.0 | 0.0 | 0.0 | 0.0 | 0.0 | 0.0 | 0.0 | 0.0 |
| Processed Animal Protein, Pork Origin | 0.0 | 0.0 | 0.0 | 0.0 | 0.0 | 0.0 | 0.0 | 0.0 | 0.0 | 0.0 | 0.0 | 0.0 | 0.0 | 0.0 | 0.0 | 0.0 | 0.0 | 0.0 | 0.0 | 0.0 | 0.0 | 0.0 | 0.0 | 0.0 | 0.5 | 0.5 |
| Fat/Oil, Palm Oil, Chemically Refined | 0.0 | 0.0 | 0.0 | 0.0 | 0.0 | 0.0 | 0.0 | 0.0 | 0.0 | 0.0 | 0.0 | 0.0 | 0.0 | 0.0 | 0.0 | 0.0 | 0.0 | 0.0 | 0.0 | 0.0 | 0.0 | 0.0 | 0.0 | 0.0 | 1.1 | 1.1 |
| Salt, NaCl | 0.0 | 0.0 | 0.0 | 0.0 | 0.0 | 0.0 | 0.0 | 0.0 | 0.0 | 0.0 | 0.0 | 0.0 | 0.0 | 0.0 | 0.0 | 0.0 | 0.0 | 0.0 | 0.0 | 0.0 | 0.0 | 0.0 | 0.0 | 0.0 | 0.3 | 0.3 |
| L-Lysine HCL | 0.0 | 0.0 | 0.0 | 0.0 | 0.0 | 0.0 | 0.0 | 0.0 | 0.0 | 0.0 | 0.0 | 0.0 | 0.0 | 0.0 | 0.0 | 0.0 | 0.0 | 0.0 | 0.0 | 0.0 | 0.0 | 0.0 | 0.0 | 0.0 | 0.1 | 0.1 |

Table SF3 Carbon footprint (g CO_2_ eq/kg feed) of different diets for three laying hen breeds.

| Item | Diet code | | | | | | | | | | | | | |
| --- | --- | --- | --- | --- | --- | --- | --- | --- | --- | --- | --- | --- | --- | --- |
|  | 151914 | 151915 | 151910 | 151911 | 151912 | 163201 | 163221 | 163231 | 163241 | 151030 | 151050 | 151100 | 151200 | 170000 |
| White |  |  |  |  |  |  |  |  |  |  |  |  |  |  |
| Land use change | 719 | 611 | 559 | 431 | 163 | 362 | 459 | 430 | 454 | 680 | 619 | 454 | 194 | 435 |
| Feed production | 605 | 578 | 584 | 575 | 553 | 554 | 550 | 542 | 536 | 580 | 573 | 572 | 558 | 542 |
| Total | 1,324 | 1,189 | 1,143 | 1,006 | 716 | 916 | 1,009 | 972 | 990 | 1,260 | 1,192 | 1,026 | 752 | 977 |
|  |  |  |  |  |  |  |  |  |  |  |  |  |  |  |
|  | 151914 | 151915 | 151916 | 151917 | 163202 | 163222 | 163232 | 163242 | 151010 | 151100 | 151200 | 170000 |  |  |
| Brown |  |  |  |  |  |  |  |  |  |  |  |  |  |  |
| Land use change | 719 | 611 | 491 | 237 | 247 | 465 | 445 | 435 | 680 | 454 | 194 | 435 |  |  |
| Feed production | 605 | 578 | 567 | 546 | 541 | 548 | 519 | 507 | 580 | 572 | 558 | 542 |  |  |
| Total | 1,324 | 1,189 | 1,058 | 783 | 788 | 1,012 | 964 | 942 | 1,260 | 1,026 | 752 | 977 |  |  |

## Energy on farm

As it was explained in the manuscript, the energy consumption on the poultry farm (lighting, ventilation, heating, etc.) was based on Kwantitatieve Informatie voor de Nederlandse Veehouderij (KWIN) 2024-2025 (KWIN, 2024). For the rearing period (assuming litter housing), the electricity consumption and the heating cost per 100 chicken was €13 and €8, respectively. For the laying period, the electricity consumption per 100 chicken was €39 (assuming aviary housing). Since the laying period of parent stocks is shorter, this value was adjusted accordingly. The energy consumption in the hatchery was €12.5 per 1,000 eggs. Subsequently, the total kWh of electricity and the m³ of natural gas were calculated using energy prices (0.14 €/kWh for electricity and 0.62 €/m³ for gas) based on KWIN (2024).

## Manure storage on the farm

To calculate the emissions of manure management, IPCC (2019) and the Dutch National Inventory Report (RIVM, 2024) were followed and where data was available, IPCC calculation method was adapted to the Dutch situation. The following equations were applied for the calculation of the direct and indirect N_2_O emissions (IPCC, 2019; Van Bruggen, et al., 2023):

| $N_{2}O_{Direct}=N_{excreted}\times{EF}_{N2O}\times\frac{44}{28}$ | Eq. 1 |
| --- | --- |
| $N_{2}O_{Indirect}=\left( {NH}_{3}-N+{NO}_{x}-N \right)\times{EF}_{NH3NOx}\times\frac{44}{28}$ | Eq. 2 |

where *N₂O_Direct_* and *N₂O_Indirect_* are the direct and indirect N_2_O emissions of excreted manure in kg N₂O, *N_excreted_* denotes the total excreted nitrogen (N) in kg N, *EF_N₂O_* shows the direct emission factor (0.001 kg N₂O-N/kg N-excretion) (IPCC, 2019), *EF_NH₃NOₓ_* is the emission factor of NH₃ and NOₓ as a result of volatilization (0.010 kg N₂O-N/kg N-Volatilized) (IPCC, 2019). *NH₃-N* and *NOₓ-N* are the quantities of NH₃ and NOₓ that are released from manure, respectively in kg NH₃-N and kg NOₓ-N. The *44/28* is the conversion factors from kg N₂O-N to kg N₂O.

The NH₃ emissions were calculated on the basis of Van Bruggen, Bannink, Bleeker, Bussink, van Dooren, Groenestein, Huijsmans, Kros, Lagerwerf and Oltmer (2023) (Eq. 3) and with the help of the amount of total ammonia nitrogen (TAN) and EF_NH3_-N. TAN was calculated by multiplying the N intake from feed by N digestibility (given by producer) and then subtracting N retention (Eq. 4). NO_x_-N was calculated using Eq. 5.

| ${NH}_{3}-N=TAN\times{EF}_{NH3}$ | Eq. 3 |
| --- | --- |
| $TAN=N_{Intake}\times N_{Digestibility coefficient}-N_{Retention}$ | Eq. 4 |
| ${NO}_{x}-N=N_{excreted}\times{EF}_{NOx}$ | Eq. 5 |

where *NH_3_-N* is the quantity of NH_3_ in kg NH_3_-N, *TAN* shows the quantities of TAN in kg TAN, *N_Intake_* is the total N intake from feed in kg N/kg feed. *N_Digestibility coefficient_* illustrates N digestibility coefficient of the feed in %. *EF_NH3_* is the emission factor of NH3 equal to 25.8 and 16.9 %NH₃-N/kg TAN for laying hens <18 weeks and >18 weeks (Van Bruggen, Bannink, Bleeker, Bussink, van Dooren, Groenestein, Huijsmans, Kros, Lagerwerf and Oltmer, 2023). *N_Retention_* denotes the amount of N retained in the animal body. N_Retention_ values ​​were 25.8, 28 and 28 g N/kg live weight for a day-old chicks, laying hens <18 weeks and >18 weeks based on (RVO, 2025). EF_NOx_ is the emission factor in %NO_X_-N/kg Total N (0.1 (Van Bruggen, Bannink, Bleeker, Bussink, van Dooren, Groenestein, Huijsmans, Kros, Lagerwerf and Oltmer, 2023)).

To calculate CH_4_ emissions from manure, IPCC (2019) method used as follows:

| ${CH}_{4}=VS\times B_{0}\times MCF\times\rho$ | Eq. 6 |
| --- | --- |

where *CH_4_* is the methane emissions from manure in kg CH_4_. *VS* is the amount of volatile solid excreted annually in kg VS/year, *B_0_* indicates the maximum amount of CH_4_ in VS which is 0.34 m³/kg VS (Van Bruggen, Bannink, Bleeker, Bussink, van Dooren, Groenestein, Huijsmans, Kros, Lagerwerf and Oltmer, 2023). *MCF* is the methane conversion factor which is the fraction of the maximum methane potential that can be released. MCF was supposed to be 1.5% (IPCC, 2019). *ρ* is the CH4 density and was considered as 0.67 kg/m³ CH4. VS was calculated based on the digestibility of the food in combination with the feed intake. Due to missing data on digestibility, VS could not be calculated. However, it is known that the feed intake differs between breeds. By assuming that there is no difference in feed digestibility, a difference in VS per breed can be estimated. This was done with the help of the national average VS production values ​​from Van Bruggen, Bannink, Bleeker, Bussink, van Dooren, Groenestein, Huijsmans, Kros, Lagerwerf and Oltmer (2023), 4.5 and 8.4 kg VS/chicken/year for chickens <18 weeks and >18 weeks, respectively. Subsequently, the annual VS production was converted to kg VS/kg feed intake and used in the further calculations.

# RESULTS OF GREENHOUSE GAS EMISSIONS

The results of GHG analysis for the rearing period of parent stocks phase for white and brown breeds per hen are shown in Table SF4. As it is seen, feed production and LUC contributed most to the total GHG emissions, followed by energy and manure management. Comparison across breeds showed the higher GHG emissions for brown breed (7.00 kg CO_2_eq/hen) than white (6.35 kg CO_2_eq/hen).

Table SF4 Greenhouse gas emissions per delivered hen (kg CO2eq/hen) at the end of rearing period of the parents stock for white and brown breeds.

| Item | Unit | White | Brown |
| --- | --- | --- | --- |
| Manure management | kg CO_2_eq/hen | 0.317 | 0.327 |
| Feed production | kg CO_2_eq/hen | 3.275 | 3.410 |
| Land use change | kg CO_2_eq/hen | 1.810 | 2.324 |
| Material and energy use | kg CO_2_eq/hen | 0.953 | 0.937 |
| Total | kg CO_2_eq/hen | 6.354 | 6.998 |

The results of GHG emissions per delivered hen during the rearing period of hens for white and brown breeds for systems with and without No-Culling Male Chicks are shown in Table SF5. The feed production and LUC (feed) were the main sources of GHG emissions and followed by the emissions associated with the production of reared chickens, manure management and material and energy use.

The breed comparison showed a higher total GHG emissions for brown (8.64 kg CO_2_eq/hen) than white (7.66 kg CO_2_eq/hen) for a system without NCMC. Same trend was seen for a system with NCC.

Tabel SF5 Greenhouse gas emissions per kg delivered hen (kg CO2eq/hen) at the end the rearing period of hens for white and brown breeds with and without No-Culling Male Chicks.

| Item | Unit | With No-Culling Male Chicks | | Without No-Culling Male Chicks | |
| --- | --- | --- | --- | --- | --- |
|  |  | White | Brown | White | Brown |
| Hatchery (day-old chick) | kg CO2eq/hen | 1.068 | 0.793 | 0.540 | 0.509 |
| Manure management | kg CO2eq/hen | 0.347 | 0.378 | 0.347 | 0.378 |
| Feed production | kg CO2eq/hen | 3.832 | 4.506 | 3.826 | 4.397 |
| Land use change | kg CO2eq/hen | 1.934 | 2.411 | 1.932 | 2.352 |
| Material and energy use | kg CO2eq/hen | 1.013 | 1.024 | 1.011 | 0.999 |
| Total | kg CO2eq/hen | 8.194 | 9.110 | 7.656 | 8.635 |

# APPLICATION OF DIFFERENT ALLOCATION METHODS

As it has been explained in the manuscript, various allocation methods namely economic, mass, protein-based and biophysical allocation methods were assessed. The impacts of different allocation methods on CF of market egg are presented in the manuscript. To provide more detail information, the allocation factors for each method are presented for white and brown breeds in Figure SF1-2. There was small difference between allocation factors for no, economic and mass allocation for white breed and more than 99% of total GHG emissions were allocated to egg, while the allocation factor for protein-based and biophysical allocation methods varied between 88% and 92% (Figure SF1). The allocation factor of meat was lower for hens phase compared to parents stock phase. More meat is produced at the end of parents stock phase resulted in higher allocation factor for meat in this phase for protein-based and biophysical allocation methods.

| 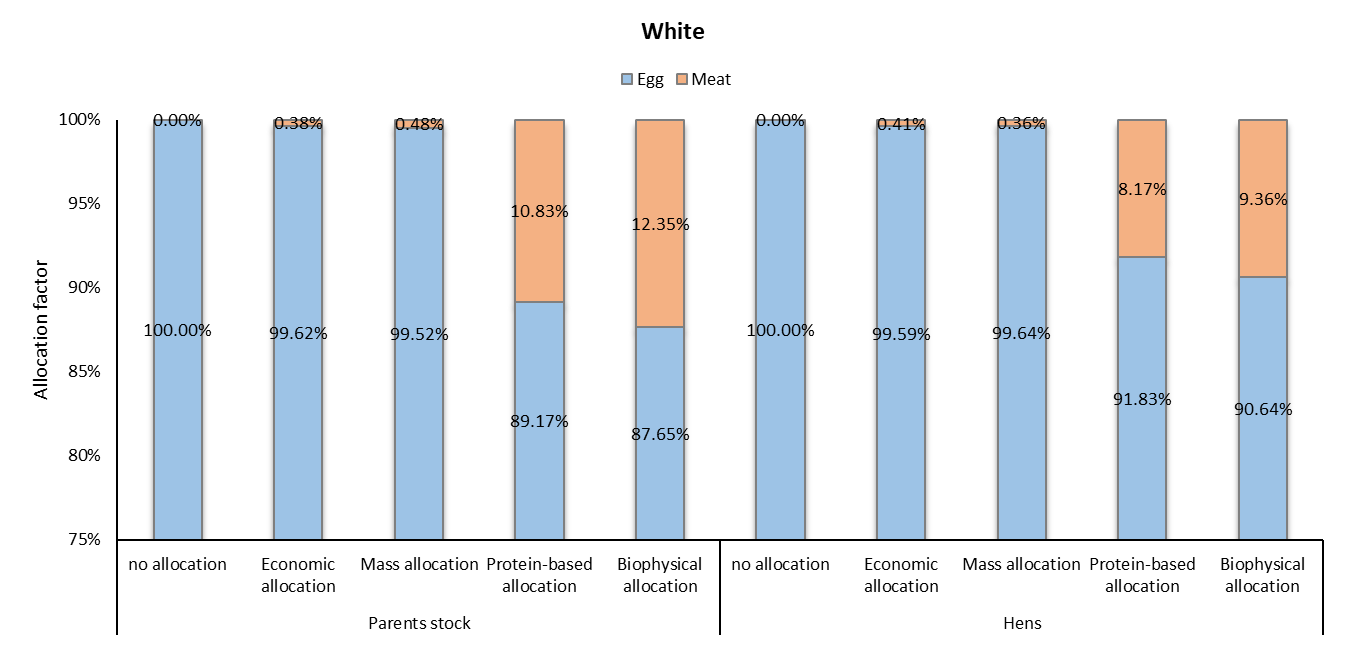 |
| --- |
| Figure SF1. Allocation factors for egg and meat for different allocation methods for white breed at parents and hens phases equipped with No-Culling Male Chicks. |

Based on obtained results, mass and economic allocation factors for market egg of brown was above 99%. The allocation factors for protein-based and biophysical allocation methods ranged from 86% to 89%. Similar to white breed, the allocation factor of meat was lower for hens phase compared to parents stock phase of brown breed. More meat is produced at the end of parents stock phase resulted in higher allocation factor for meat in this phase for protein-based and biophysical allocation methods.

| 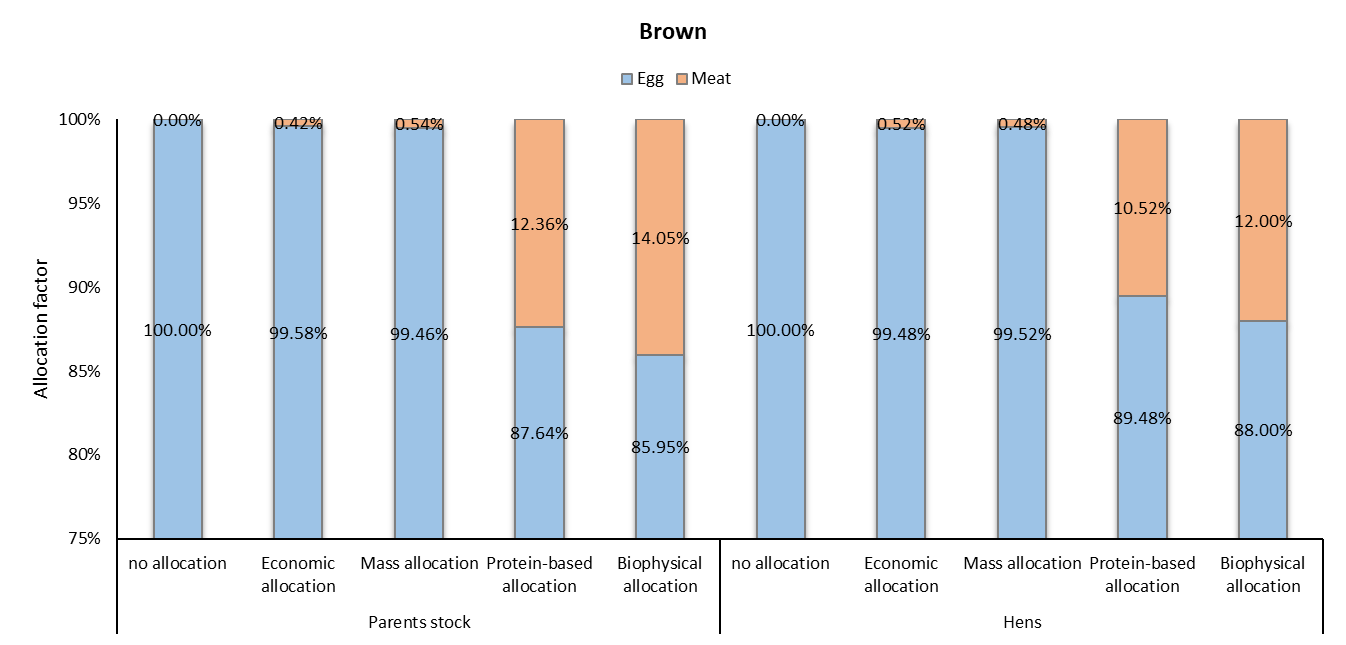 |
| --- |
| Figure SF2. Allocation factors for egg and meat for different allocation methods for brown at parents and hens phases equipped with No-Culling Male Chicks. |

The results of assessing the impact of application of different allocation methods on CF of market egg (kg CO_2_eq/egg) for White and brown are shown in Figure SF3. As it has been explained, allocation was applied at laying period of parent stocks and hens phases. In our assessment, the reference situation was no allocation, in which all GHG emissions were allocated to eggs. Therefore, the highest CFs of 0.15, and 0.17 kg CO_2_eq/egg were seen in this situation for white and brown breeds, respectively. Obtained results for white breed showed that application of economic, mass, protein-based and biophysical allocation resulted in reduction of CF of market egg (kg CO2eq/egg) by 0.3%, 1.1%, 8.2% and 9.4%, respectively. For brown breed the reduction for economic, mass , protein-based and biophysical allocation was 0.4%,1.0%, 10.5% and 12.0%, respectively.

| 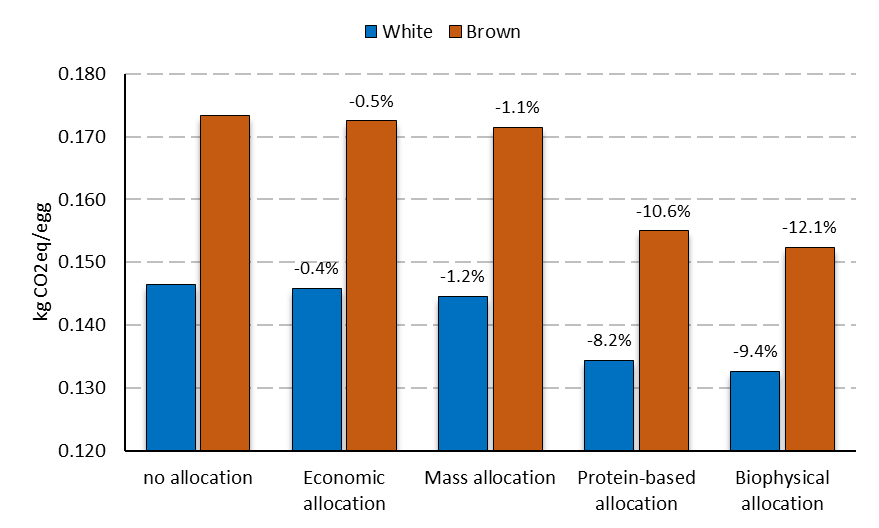 |
| --- |
| Figure SF4. Carbon footprint (kg CO_2_eq/egg) of market egg equipped with No-Culling Male Chicks using different allocation method. Percentages on top of each column indicates the difference from no allocation. |

REFERENCES

Blonk Consultants. 2022. Agri-Footprint 6.3: Environmental footprint database for agri-food products. Blonk Consultants. Retrieved from <https://www.agri-footprint.com>.

IPCC. 2019. 2019 Refinement to the 2006 IPCC Guidelines for National Greenhouse Gas Inventories, Calvo Buendia, E., Tanabe, K., Kranjc, A., Baasansuren, J., Fukuda, M., Ngarize, S., Osako, A., Pyrozhenko, Y., Shermanau, P. and Federici, S. (eds). Published: IPCC, Switzerland.

KWIN. 2024. Kwantitatieve Informatie Veehouderij 2024-2025. Wageningen Livestock Research. Retrieved from <https://shop.wur.nl/kwin/kwin-veehouderij-2024-25.html>.

RIVM. 2024. Greenhouse gas emissions in the Netherlands 1990–2022. National Inventory Report 2024. RIVM report 2024-0017.

RVO. 2025. Tabellen mest. <www.rvo.nl>.

Van Bruggen, C., A. Bannink, A. Bleeker, D. Bussink, H. van Dooren, C. Groenestein, J. Huijsmans, J. Kros, L. Lagerwerf, and K. Oltmer. 2023. Emissies Naar Lucht Uit de Landbouw Berekend met NEMA voor 1990–2021. Wettelijke Onderzoekstaken Natuur & Milieu: Wageningen, The Netherlands.
